# “I am not a number!” Opinions and preferences of people with intellectual disability about genetic healthcare

**DOI:** 10.1038/s41431-023-01282-3

**Published:** 2023-01-20

**Authors:** Iva Strnadová, Julie Loblinzk, Jackie Leach Scully, Joanne Danker, Michelle Tso, Karen-Maia Jackaman, Manjekah Dunn, Sierra Angelina Willow, Skie Sarfaraz, Vanessa Fitzgerald, Jackie Boyle, Elizabeth Emma Palmer

**Affiliations:** 1 School of Education, UNSW Sydney, Sydney, NSW Australia; 2 Disability Innovation Institute, UNSW Sydney, Sydney, NSW Australia; 3 Self-Advocacy Sydney, Sydney, NSW Australia; 4 Gonski Institute for Education, UNSW Sydney, Sydney, Australia; 5 Discipline of Paediatrics and Child Health, School of Clinical Medicine, Faculty of Medicine and Health, Sydney, NSW Australia; 6 NSW Ministry of Health, St Leonards, NSW Australia; 7 NSW Genetics of Learning Disability Service, NSW Health, Waratah, NSW Australia; 8 Sydney Children’s Hospitals Network, NSW Health, St Leonards, NSW Australia

**Keywords:** Genetic counselling, Genetic testing

## Abstract

There is limited research exploring the knowledge and experiences of genetic healthcare from the perspective of people with intellectual disability. This study, conducted in New South Wales (Australia), addresses this gap. Eighteen adults with intellectual disability and eight support people were interviewed in this inclusive research study. The transcribed interviews were analysed using inductive content analysis. The findings were discussed in a focus group with ten adults with intellectual disability and in three multi-stakeholder advisory workshops, contributing to the validity and trustworthiness of the findings. Five main themes emerged: (i) access to genetic healthcare services is inequitable, with several barriers to the informed consent process; (ii) the experiences and opinions of people with intellectual disability are variable, including frustration, exclusion and fear; (iii) genetic counselling and diagnoses can be profoundly impactful, but translating a genetic diagnosis into tailored healthcare, appropriate support, peer connections and reproductive planning faces barriers; (iv) people with intellectual disability have a high incidence of exposure to trauma and some reported that their genetic healthcare experiences were associated with further trauma; (v) recommendations for a more respectful and inclusive model of genetic healthcare. Co-designed point-of-care educational and consent resources, accompanied by tailored professional education for healthcare providers, are required to improve the equity and appropriateness of genetic healthcare for people with intellectual disability.

## Introduction

Globally, 78 million people (1% of the population) have intellectual disability [1]. Diagnostic genomic technologies are revolutionising our understanding of the underlying causes of over 50% of intellectual disability in high income countries. A genetic diagnosis can improve overall health outcomes through precision medicine, empowerment, and reproductive choice [2]. Our recent systematic literature review showed people with intellectual disability risk exclusion from these opportunities through inequitable, non-inclusive healthcare and lack of data on their experiences, opinions, and preferences. Only 7 studies asked people with intellectual disabilities about their experiences and/or preferences about genetic testing and counselling. Most participants in these studies expressed the desire to learn more about genetic conditions and genetic tests and held diverse opinions about genetic tests. Non-inclusive healthcare practices were evident, and no study was conducted in a fully inclusive manner, with co-researchers with intellectual disability as part of the team.

People with intellectual disability need meaningful opportunities to contribute to healthcare service design and delivery [3–5]. More inclusive models of care reduce existing health inequities and improve healthcare outcomes [6]. Work is required to build and sustain public confidence in genetics, including partnering with consumers to develop services that reflect their needs [7]. After discussions with New South Wales (NSW) Health, an inclusive research program was developed in partnership with multiple stakeholders from health, government, self-advocacy, rare disease, disability, and education sectors. The aim was to explore people with intellectual disabilities’ knowledge, perspectives, and experiences of genetic testing and counselling (‘genetics healthcare services’) across NSW Health.

## Materials and methods

### Participants

Convenience sampling was used to recruit 18 adults with intellectual disability and eight support people from disability organisations and services run by advocates with intellectual disability, and several genetic services across NSW Health with which the research term had existing professional relationships. We recruited adults with a mild intellectual disability and capacity to provide informed consent, as ascertained by the experienced research team, who had had genetic testing or contact with a genetic health service in NSW at any stage of their life (including as a child). The level of intellectual disability had been assessed by a healthcare professional and was provided by the genetic service or by the participant/support person themselves. Participants had a variety of genetic diagnoses including single gene and chromosomal conditions, for example Coffin–Lowry syndrome and *HUWE1*-related condition; six had no genetic diagnosis. Demographic details of participants and of support people are provided in Tables 1 and 2 respectively. Participants chose to use their own or a researcher-chosen pseudonym, or their own name.

**Table 1:** Demographics of participants and their support people.

Pseudonym/Real name	Sex	Age (years)	Age at genetic testing (years)	Relationship status	Children	Employment status	Living arrangement	Location	Interview length (hours)	Support person present	Interview format
Katarina	F	41	41	Married	5	Unemployed	House with son and friend	Regional	1:01	No	Zoom
Mason	M	56	40	Married	1 stepchild	Retired	Department of housing unit with wife	Metropolitan	1:39	No	Zoom
Milly	F	53	37	Married	1	Retired	Department of housing unit with husband	Metropolitan	1:39	No	Zoom
Wesley	M	20	3	Single	0	Part time	Group home with flat mate	Metropolitan	0:47	Yes	Zoom
Sarah	F	35	35	Married	1	Unemployed	House with family	Metropolitan	0.39	No	Zoom
Sam	M	31	At school age	Single	0	Part time	Unit by self	Metropolitan	0:33	Yes (same as for James)	Zoom
James	M	33	At school age	Engaged	0	Part time	Unit with fiancée	Metropolitan	0:33	Yes	Zoom
Jeremy	M	22	3	Single	0	Part time	House with parents	Metropolitan	0:31	No	Zoom
Aaron	M	45	10	Single	0	Part time	House with family	Metropolitan	1:46	Yes	In person
Lisa	F	28	At young age	Single	0	Volunteering	Not specified by self with support	Rural	N/A	Yes	Written response
Ian	M	41	At school age	Single	0	Part time	Care home with flat mate	Metropolitan	1:01	Yes	Zoom
Jeffrey	M	64	At young age	Dating	0	Volunteering	Department of housing by self	Metropolitan	0:18	No	In person
Richard	M	65	At young age	Divorced	3	Volunteering	Department of housing house with son	Metropolitan	0:21	No	In person
Lilian	F	23	A few years ago	Single	0	Part time	Not specified by self	Metropolitan	0:53	No	Zoom
Maria	F	56	A few years ago	Married	5	Unemployed	Department of housing house with husband and children	Regional	1:05	Yes	In person
Anton	M	18	5	Single	0	Full time	House with family	Metropolitan	0:46	Yes	Zoom
Sammy	F	31	Unsure	Dating	0	Work experience	Department of housing with family	Metropolitan	0:38	No	In person
Riley	M	26	At young age	Single	0	Studying	House with family	Metropolitan	0:35	Yes	Zoom

**Table 2:** Demographics of support people.

Pseudonym	Sex	Age	Relationship status	Children	Employment status	Living arrangements	Location	Interview length	Relationship with participant	Interviewed with participant
Martha	F	Not specified	Widowed	Not specified	Not specified	Not specified	Metropolitan	0:47	Grandmother of Wesley	Yes
Teresa	F	55	Married	3	Part-time	Family house	Metropolitan	0:34	Mother of Sam and James	Yes
Betty	F	69	Widowed	3	Retired	Family house	Metropolitan	1:46	Mother of Aaron	Yes
Jess	F	54	Married	4	Full-time	Family house	Rural	N/A	Mother of Lisa	Written response
Pat	F	85	Not specified	Not specified	Not specified	Not specified	Metropolitan	1:01	Grandmother of Ian	Yes
Gabe	M	59	Married	5	Not specified	Family house	Regional	1:05	Husband of Maria	Yes
Jackie	F	53	Married	2	Full-time	Family house	Metropolitan	0:46	Mother of Anton	Yes
Margon	F	61	Divorced	2	Unemployed	House with family	Metropolitan	0:35	Mother of Riley	Yes

### Inclusivity of research study

Our research team included two co-researchers with intellectual disability. All study materials, including participant information statement and consent forms (PISCF), were available in Standard and Easy Read English (see Appendix A).

### Interviews

The interview protocol (Appendix B) was based on our systematic review of the literature [8] and input from the multidisciplinary research team (a bioethicist, researchers in special education and disability studies, health researchers, a genetic counsellor, a clinical geneticist, and co-researchers with intellectual disability).

Two members of the research group (IS and JL) conducted the interviews via Zoom Video Communications, or, when requested, in participants’ homes. JL is a co-researcher with intellectual disability and extensive experience in inclusive research with IS. Five interviews were conducted at participants’ homes, 12 were conducted via Zoom and one participant preferred to provide a written response to the interview questions. Interviews averaged 55 minutes in length (range 21 minutes to 1 h 46 minutes).

### Data analysis

The audio-recorded interviews were transcribed and then analysed using inductive content analysis. Inductive content analysis involves the systematic coding and categorisation of data to understand participant narratives [9]. As an inductive method it prioritises meaning drawn from the dataset itself, rather a deductive approach which applies theory to understand the data. In the initial open coding stage, codes represent blocks of raw data. Two authors (IS and SC) independently open-coded one interview by breaking down the transcript and assigning a code label to each unit of meaning. After resolving any differences in the coding, SC then open coded all remaining interviews and IS checked coding accuracy. Another author (JD) then refined and clustered the codes into categories, sub-themes, and themes. Themes were confirmed through triangulation of interview quotes, codes, memos, and frequency of occurrences. Mind-maps of the codes, categories, sub-themes, and themes were also created using the visual collaborative platform MIRO to facilitate accessible visual representation of the data. The entire research team (i.e., academic researchers and co-researchers with intellectual disability) then provided feedback.

### Validity and trustworthiness

**Fig. 1:**
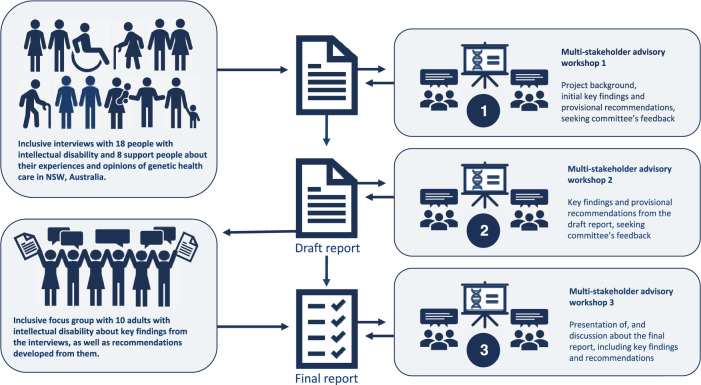
Overview of Project. Infographic of the research program protocol.

The findings were discussed in a focus group with ten adults with intellectual disability from a self-advocacy organization, and three multi-stakeholder advisory workshops with 25 people from health, disability, rare disease, and government organizations (Fig. 1). Consultation with and affirmation from individuals with professional and lived experience contributed to the validity and trustworthiness of the findings.

## Results

**Fig. 2:**
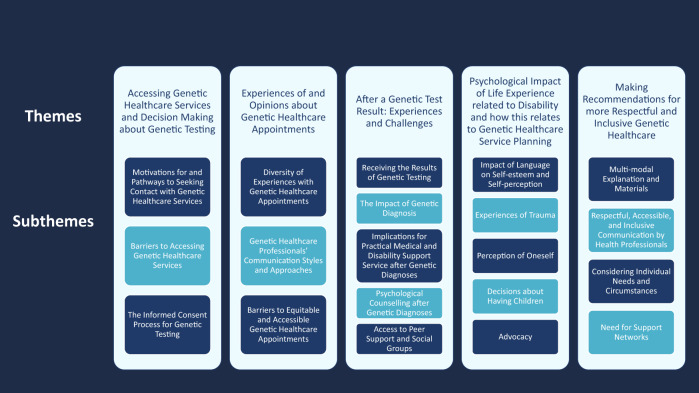
Graphic summary of inductive content analysis. Overview of the themes and subthemes that emerged from data analysis.

Five main themes emerged from the interviews (Fig. 2).

### Theme 1: Accessing genetic healthcare services and decision making about genetic testing

#### Motivations for and pathways to seeking contact with genetic healthcare services

Many people with intellectual disability did not actively choose to attend a genetic health service. Instead, contact was initiated by family members, doctors, or researchers, often motivated by the needs of a parent or sibling wanting to know if intellectual disability in the family was heritable. Many participants seemed uncertain about why they were referred:

“…they want to do research on me, for my doctor …” (Lilian).

Two participants sought genetic healthcare to increase their knowledge of their condition or for information to guide their reproductive planning prior to marriage.

Most participants could identify potential benefits of genetic testing and diagnosis (understanding more about a condition, ‘ruling out’ certain conditions, and accessing appropriate assistance, funding, and reproductive planning), although none specifically mentioned the benefits in guiding health surveillance or treatments.

“…I feel that it would be helpful for families … especially the young ones these days that have had children, babies that have disabilities, to help them learn a bit more…” (Milly).

Notwithstanding these benefits of genetic testing, participants felt that seeing a genetic healthcare service and/or have genetic testing should be a personal choice. Several reported having family members who were reluctant to get genetic testing. Aaron and his mother Betty acknowledged that others may hesitate due to a feeling of intrusion “and also a bit of denial that there could be a problem in their family”, while Richard said he viewed genetic testing as negative and preferred to “look at the positive side of [having intellectual disability] not the negative side”.

#### Barriers to accessing genetic healthcare services

Participants identified several barriers, including their own and their families’, specialists’, or primary healthcare providers’ lack of knowledge about genetic health services, difficulties finding a local service or counsellor, and long wait times.

#### The informed consent process for genetic testing

Participants had variable experiences and wanted more choice over the consent process, appointment settings, inclusion of support people, and the way that information and forms were presented to them. Some participants felt included in the process and comfortable with their decisions:

“They asked me, can we do it… I said, ‘If you want to. I’ve got nothing to hide. Go for it.’” (Maria)

Others felt excluded because the process was rushed, not explained accessibly, and/or consent was sought from their carers. Lilian disapproved of the hurried pace of the appointment: “they just quickly just, yeah, yeah, go and do this, let’s do it …”.

### Theme 2: Experiences of and opinions about genetic healthcare appointments

#### Diversity of experiences with genetic healthcare appointments

Although some people had positive experiences with genetic healthcare services, others felt confused, frustrated, unsupported, excluded, or frightened. Jackie, a support person, noted, “I just remember going home feeling happy about the interaction with them” whereas Aaron recalled his testing experience with bewilderment: “I was sitting there, dumbfounded, like that”. Lilian suggested that a support person could help by rephrasing complicated questions and “can talk for you when you don’t understand…. There always needs to be an advocate, like my mum.” There were some participants whose parents were not invited to attend. Jeremy appreciated that some teams let him choose whether to have his parents there.

#### Genetic healthcare professionals’ communication styles and approaches

Participants had experiences ranging from very negative: “They [medical professionals] don’t treat you like a person. They treat you like an ‘it’.” (Richard) to more positive: “We had a really good experience the whole way through. Very supportive.” (Teresa, support person). Participants reflected that “genetics is very hard to understand” (Mason and Milly) and wished they had more genetics education at school.

One communication barrier was inaccessible information: “It wasn’t Easy Read. … it’s very hard to read the form…? Even my mum found it hard to read…” (Lilian).

#### Barriers to equitable and accessible genetic healthcare appointments

Participants identified several barriers, including logistics, difficulties navigating the health system, the perceived invasiveness of genetic counselling and testing, a lack of inclusive practice by health professionals and service providers, communication issues, and rushed consultations. Martha noted that Wesley needed to have “government funding or whatever” since he needs “the whole lot of genomic testing”. Blood-taking was concerning for several: “… people with intellectual disability don’t like having their blood drawn out because they’re scared…. They get worried.” (Lilian)

### Theme 3: After a genetic test result: experiences and challenges

#### Receiving the results of genetic testing

Not all participants remembered being given the results of their genetic testing. Support people who were present often acted as ‘gatekeepers’, deciding that their child(ren) did not need detailed information on their genetic diagnosis.

“I didn’t continue trying to explain. Her father and I understood the results and that is enough…. She didn’t, and won’t, understand the technical language and explanation of her results”. (Jess, support person).

Participants reported inconsistency in communication or sharing of results. Most experienced long wait periods between testing and results without explanation or preparation for the wait. Katarina said:

“… they’ll do a blood test that will take four weeks to a month to come back, that gets sent to [another state in Australia]. In that meantime, I was stressing the whole time!”

#### The impact of a genetic diagnosis

Participants reported many emotions after their genetic diagnosis, from acceptance and relief to depression and anger. Jeremy described his long journey to accepting his diagnosis, “I really struggled with that, but then I kind of grew up more and more, I kind of accepted it more”. Katarina said “I feel like I’m not normal now. And I’ve told people about it, and they’re my friends and family and they don’t mean to pick on me about it, but they look like, ‘You’re just a retard. You’re not all there now’”. Two participants shared that they had considered suicide after their diagnosis, with Mason reflecting, “I came to a point when I found out, … I had contemplated on suicide.”

Commonly, participants and their carers had ongoing difficulties recalling the name of the diagnosed genetic condition, especially if based on a gene name. The use of a combination of letters and numbers for a condition was also sometimes upsetting: “I’m not a number!” (Maria).

#### Implications for practical medical and disability support service after genetic diagnoses

After a genetic diagnosis, many participants either had not been told what specialist medical care and disability support was required or encountered barriers in accessing such care. Katarina described her difficulties accessing NDIS (National Disability Insurance Scheme) support as “there is no NDIS for chromosome 16”.

#### Psychological counselling after genetic diagnoses

Most participants stressed the need for adequate preparation before a genetic diagnosis and for psychological support afterwards. Some participants found the genetic health services exemplary: they valued the supportive genetic counsellors, and the team approach of the service meant that even if their key counsellor was unavailable other team members had access to their clinical records and could provide ongoing support.

#### Access to peer support and social groups

No participants recalled their genetic or primary healthcare teams linking them with support groups for their own genetic condition. Many wanted to connect with other people with the same genetic diagnosis, ideally in person, but otherwise via video-conference or social media. Katarina stated that she wanted to join a support group: “and have people all over the world explain what they’re going through. How they’re dealing with it.”

### Theme 4: Psychological impact of life experience related to disability and how this relates to genetic healthcare service planning

#### Impact of language on self-esteem and self-perception

All participants with intellectual disability referred to themselves as being ‘not normal’ or having ‘something wrong with me’. They had repeatedly encountered deficit language in other healthcare, schooling, and employment settings, and from their family and peers. This meant that when words such as ‘abnormality’, ‘faulty gene’, ‘mutation’, or ‘disorder’ were used at genetics appointments, the negative connotations were internalised by the participants.

“…they did show me, yes, you have got the bad X [referring to X chromosome] on Mum’s side of the family, like that, and I knew I wasn’t normal to other – I knew I was missing, some part of my brain has gone missing.” (Aaron)

The stigma of having intellectual disability affected every aspect of the participants’ lives.

“I’m never going to be able to read and write properly, or spell (crying). I’m never going to be able to learn that. And that breaks me.” (Katarina)

#### Experiences of trauma

Questions about abuse were not included in the interview guide, but were spontaneously brought up by the participants, for example when discussing the lack of choices before and during appointment and reproductive planning. Katarina had no choice over the gender of her clinical geneticist and said: “…I get scared of males, I’m petrified of males, because I was sexually assaulted and abused…. And raped fifteen times.”

Participants emphasised the importance of being asked who they would prefer as their support person, rather than assuming this would be a parent, given several participants stated that perpetuators of abuse included their family and carers. For example, Sammy highlighted financial abuse by her parents who were “currently saving up our money, my sister’s and my money. So, in a way, they’re sort of taking advantage of us, as well.”

The repeated trauma is a sad reflection of reality and was usually not disclosed by the participant to their genetics healthcare teams, who remained unaware of this important context for their patients. Moreover, that healthcare professionals’ own attitudes and behaviour can be traumatising was confirmed by the focus group with people with intellectual disability. One participant stated: “Doctors sometimes treat you as a number.” Another said: “Doctors know that people with intellectual disability will not question them…and they abuse it.”

#### Perception of oneself

Such experiences heavily impacted the way participants perceived themselves. Negative self-perceptions caused by a life history of stigma, trauma, and rejection by society and often also by family members, contributed to poor mental health and low self-esteem, and the sense of being a burden on their families or society.

Others were able to see their own worth and the value people with intellectual disability bring to society. Many listed their achievements, such as driving a car or strengths in arts, and said they appreciated when these achievements were acknowledged by their healthcare teams. “When I first told … the team what I accomplished throughout the years. They were amazed. They were speechless.” (Mason).

#### Decisions about having children

This history of trauma also impacted decisions about pregnancy. Participants had a wide range of attitudes towards family planning and genetic testing in pregnancy; many felt that the choice of whether to have children was a human rights issue.

Several had or planned for children. Some reached this decision after careful consideration, including their own experience of intellectual disability and the related societal stigma. They felt that their lived experience would enable them to be good parents, especially if their children had intellectual disability.

Others were less certain about their own reproductive future, and some had decided not to have children after considering the impact on their lifestyles and the responsibility involved. At times there was an obvious tension between the participant’s wishes, and their parents’/grandparents’ opinions. “Mum was angry because I got pregnant, because she didn’t want another disabled child, and she knew that I might not have been able to cope. … [But] when my first sister, my elder sister, got pregnant, she was happy…” (Milly).

Some participants advocated for genetic testing in the process of family planning. In this context it was not perceived as eugenic but as an aid to informed decision making, giving people time to prepare to be parents of a child with a disability. As Jeremy summarised it: “As I’ve grown older, I do want to have kids, and if they’re autistic, that’s fine… because I lived with it, and I can support them.”

#### Advocacy

Participants raised the importance of self-advocacy and the need to be aware of patient healthcare rights in the period after accessing genetic healthcare services. Mason said: “Everyone’s got disability. When they find out they’ve got a condition like I’ve got, I feel that they need to come up front and speak up, and not shut down”. Milly agreed, declaring that without the ability to self-advocate and “speak your mind…you won’t get anywhere in life”.

Several participants spoke eloquently of more positive experiences of self-advocacy [10, 11], including in healthcare. They highlighted the importance of support people who were advocates and of self-advocacy in driving positive life experiences.

### Theme 5

#### Making recommendations for more respectful and inclusive genetic healthcare

Participants consistently identified solutions to improve the delivery of equitable and respectful genetic healthcare, also relevant to all healthcare, which can be presented as the following recommendations.

##### Multi-modal explanation and materials

Most agreed that information on genetic healthcare should be presented in multimodal ways. “I’ve always much preferred visuals over writing or things like that” (Jeremy). Aaron suggested developing cartoon-style resources to make genetic healthcare information accessible: “… even if it’s just a little video, or pictures, or words, …like a cartoon character, or someone famous they know.”

Mason and Milly suggested developing an Easy Read information package for patients with intellectual disability, “Simple, like [my genetic counsellor] did with this information for us.”

##### Respectful, accessible, and inclusive communication by health professionals

Many recommendations concerned genetic healthcare professionals’ communication with patients with intellectual disability. Participants emphasised the importance of medical professionals introducing themselves and talking directly to them: “Say hi, and what do you do for us, and introduce first…”(Lilly). As Sammy stressed: “Everyone deserves respect.” It also meant not to being talked down to or treated like a child. “But babying people, like, I’ve had that all my life…Like, no, no, no!” (Riley).

Most participants complained that doctors use complicated medical terms: “…don’t just have the big words. (…) break the words up for us.” (Katrina). Participants highlighted the need for enough time for quality communication: “…give me time to think, so as I can listen…listen to them before they reply” (Ian).

##### Considering individual needs and circumstances

As well as a calm and welcoming environment in the medical setting, another recommendation was that genetic healthcare be in easily accessible locations: “For us to drive there, it was like two hours there and two hours home … That’s four hours just for an hour’s appointment” (Sarah). Wait times for test results should also be shorter.

##### Need for support networks

Participants recommended access to genetic support networks. Milly complained: “Where is a group for [name of the syndrome]? They should put that together. I think there should be … support group to help the community that has this condition, to understand everyone’s knowledge of what they have gone through, and help the families, help the parents to understand as well.” For Lillian such a group would be an important source of connection “…you can communicate, make friends, …socialise… Just to … know what you have, know that you can have that relationship…”

## Discussion

### Importance of the study

This inclusive study fills an identified knowledge gap in how people with intellectual disability experience genetic healthcare services (9). Our study supports the findings of our systematic literature review (9) that people with intellectual disability have both the desire and the capability to learn more about genetic testing and their own genetic conditions. We also found a range of opinions and preferences, and very diverse experiences of genetic healthcare. Critically, the participants suggested many recommendations for improving genetic healthcare, and indeed healthcare more generally. This highlights the importance of careful engagement with genetic service end users to thoroughly understand not only the barriers to respectful, accessible, and inclusive genetic healthcare, but also powerful enablers.

Combining the recommendations of participants, the focus group, and advisory workshops, a list of recommendations was compiled (Table 3) to improve the model of genetic healthcare for people with intellectual disability.

**Table 3:** Recommendations for an improved model of care.

	Summary of recommendations
Recommendation 1	A suite of accessible point-of-care resources for genetic healthcare education and awareness should be available.
Recommendation 2	Resources should be co-produced with people with intellectual disability to improve the accessibility of the genetic informed consent process.
Recommendation 3	Education for healthcare providers, including for genetics and non-genetics professionals such as general practitioners, should be tailored to improve knowledge and skills in, and the motivation to apply, best practice genetics healthcare for people with intellectual disability, including trauma-informed care, cultural safety, and awareness.
Recommendation 4	In view of the complex impacts of genetic diagnoses, national and international recommendations for best practice care in clinical genetics for people with intellectual disability should be developed.
Recommendation 5	A customisable Easy Read communications folder should be co-designed to help genetic healthcare providers translate a genetic diagnosis into improved integrated care for all people with intellectual disability.

Participants had limited knowledge of the genetic testing process or genetic terminology. This highlights the need for relevant information before and during a genetic healthcare appointment to maximise understanding and capacity for shared medical decision-making. Primary healthcare providers were identified as potential key coordinators who could raise awareness of genetics and facilitate shared decision-making [12].

Our study also identified a need for comprehensive patient support after genetic healthcare, in line with current recommendations for all rare disease patients [13, 14]. This included ‘checking-in’ after a diagnosis, ensuring access to understandable resources on genetic conditions, facilitating psychological and practical supports such as tailored disability funding and allied health, educational and/or workforce support, and providing information on peer-support groups or networks. These four areas (educational, psychological, practical, and social support) are also highlighted in the rare and complex disease literature [15, 16].

A key facilitator of equitable genetic healthcare is respectful and accessible communication between healthcare staff and people with intellectual disability. Appropriate communication is critical at all points of genetic healthcare – from referral, first meeting with a genetic counsellor, to genetic testing and post-appointment care [17]. People with intellectual disability should be treated with dignity and addressed directly [18, 19]. Accessible communication includes the use of plain English, avoiding medical jargon, checking for understanding, speaking slowly, and allowing enough time in the consultation to build rapport with the person with intellectual disability: all points in line with studies in other areas of healthcare [20]. Moreover, all printed information should be provided in Easy Read [21, 22]. Such appropriate communication is required under local and international legislation, as reasonable adjustments to ensure information is presented in an understandable way [3, 4]. However, despite Australia being a signatory to the United Nations Convention on the Rights of Persons with Disabilities [3], and having a Charter of Health Care Rights [4], it is evident that reasonable adjustments are not universally made either in genetic healthcare or indeed healthcare more generally.

We were unable to find openly accessible genetic resources designed for people with intellectual disability anywhere in the world. Current training for genetics healthcare professionals (Clinical Geneticists and Genetic Counsellors) in Australia does not specifically address how to include people with intellectual disability in genetic healthcare or provide accessible genetic counselling. Improving healthcare provider education to shift attitudes and improve skills in person-centred communication should be prioritised [8].

Given how commonly people with intellectual disability have experienced trauma, and how this trauma affects their healthcare experiences, it is important to deliver genetic healthcare in a trauma-informed manner [23]. Trauma-informed care is “an approach to service delivery based on an understanding of the ways trauma affects people’s lives, their service needs and service usage” [24]. Trauma-informed best practice genetic healthcare would make appropriate adjustments in line with a person’s disability, build rapport, and prepare people for what happens next in an appointment, actively eliciting and facilitating their preferences and choices so that they feel safe and respected [23, 25, 26]. Our participants’ distressing experiences with healthcare providers are in line with other studies highlighting that healthcare professionals often exclude people with intellectual disability from consultations, misattribute all health issues to their disability, and sometimes display unprofessional behaviour [27–29].

### Limitations of the study and areas of future research

Although we recruited participants from metropolitan, rural and regional settings, this study was confined to a single state in Australia. Future inclusive qualitative studies should cover other states in Australia, and research led by Indigenous researchers is required to assess the needs of Indigenous people with intellectual disability and their families, carers, and community [30]. Effective inclusive qualitative research such as arts-based methods (e.g. Photovoice and body mapping) should be used to gather the opinions and preferences of children and people with higher support needs [5, 8, 31–33].

A major focus of future research should be the co-production of solutions to the barriers and challenges identified by this qualitative study, including co-designed standards of genetic healthcare for people with intellectual disability, clinician educational resources and a suite of accessible multimodal resources to support genetic counselling and the informed consent process.

## Conclusions

Non-inclusive models of care can compromise the quality of healthcare for people with intellectual disability and contribute significantly to their recognised poor health outcomes [6, 34]. Many people with intellectual disability actively distrust the health service, having previously faced discrimination and marginalisation [35, 36]. Our study took an inclusive approach to build trust and confidence and allow an in-depth exploration of our participants’ experiences and health needs and provide them with a meaningful opportunity to inform changes in healthcare services [5, 37]. We collaborated with a range of relevant stakeholders to maximise the relevance of our findings and ensure future successful implementation into our genetic healthcare model.

The co-produced recommendations highlight several opportunities for improving awareness of and equity in access to genetic health services. They also provide suggestions for ‘translating’ a genetic diagnosis into more tailored and holistic medical care, and broader multi-faceted support. These recommendations are relevant not only to those with intellectual disability, but embody key person-centred healthcare principles applicable to the 300 million people living with rare diseases globally [38].

## Supplementary information

Appendix A

Appendix B

## Data Availability

The datasets generated during and/or analysed during the current study are not publicly available due the individually rare nature of the genetic diagnoses which could potentially mean individual participants were identifiable. However aggregate, non-identifying, data is available from the corresponding author on reasonable request.
