# Supplementary material for: “I am not a number!” Opinions and preferences of people with intellectual disability about genetic healthcare
Source: Eur J Hum Genet. 2023 Jan 20;31(9):1057–65. doi: 10.1038/s41431-023-01282-3 (PMC10474088; doi:10.1038/s41431-023-01282-3)
Supplement: Supplementary file 1 — Appendix A [file 41431_2023_1282_MOESM1_ESM.docx]

**Appendix A:
Standard and Easy Read English participant information statement and consent forms**

1. **What is the research study about?**

You are invited to take part in this research study. The research study aims to understand the opinions and experiences of people with intellectual disability about genetic counselling and /or testing. This is to help us provide guidance to NSW Health, doctors, and genetic counsellors in how the model of care can be improved for people with intellectual disability.

1. **Who is conducting this research?**

The study is being carried out by the following researchers:

Dr Elizabeth Emma Palmer School of Women’s and Children’s Health, UNSW

Professor Iva Strnadová School of Education, Arts, Design, and Architecture, UNSW, and Disability Innovation Institute, UNSW

Professor Jackie Leach Scully Disability Innovation Institute, UNSW

Ms Julie Loblinzk School of Education, Arts, Design, and Architecture, UNSW

Ms Jackie Boyle Genetics of Learning Disability (GOLD) Service, Hunter Genetics, Hunter New England Local Health District

**Research Funder:** This research is being funded NSW Department of Health

1. **Inclusion/Exclusion Criteria**

Before you decide to participate in this research study, we need to ensure that it is ok for you to take part. The research study is looking recruit people who meet the following criteria:

1. 18 years or older,
2. People with an intellectual disability (mild to moderate),
3. People who have had experience with genetic counselling and/ or testing,
4. People currently living in NSW,
5. People who have the capacity to consent participate in this study.

Participants who meet the following criteria will be excluded from the study:

1. Under the age of 18 years,

2. With a severe or profound intellectual disability,

3. Without the capacity to consent to participate in the study,

4. People who do not have experience with genetic counselling and or testing,

5. People who currently do not live in NSW.

1. **Do I have to take part in this research study?**

Participation in this research study is voluntary. If you do not want to take part, you do not have to. If you decide to take part and later change your mind, you are free to withdraw from the study at any stage.

If you decide you want to take part in the research study, you will be asked to:

- Read the information carefully (ask questions if necessary);
- Sign and return the consent form if you decide to participate in the study;
- Take a copy of this form with you to keep.

1. **What does participation in this research require, and are there any risks involved?**

If you agree to participate you will be asked to complete either an interview or focus group.

**Interview:** You will be offered the opportunity of an interview, and you will be asked questions about your opinions and experiences about genetic counselling and / or testing. You can choose between taking part face-to-face, online (i.e., video-conferencing, using a platform of your choice), or via phone. The interview will take place at an agreed location close to where you live and will take approximately 30 minutes to 1 hour. With your permission the research team would like to audio and video record the interview. If you do not wish to be recorded but you would like to participate you advise the research team and written notes will be taken. You may also be asked if you would like to join in a **focus group.**

**Focus Group:** All focus group sessions will take place either online or face-to-face in a community centre close to where you live and will take approximately 1 hour. During the focus group you will be asked questions about your opinions and or experiences with genetic testing and or counselling and/ or with a draft report we will have prepared including recommendations for health providers and government regarding genetic counselling and testing.

With your permission the research team would like to audio and or /video record the interview.

If you decide to participate in the focus group, your comments along with other participants will be recorded during the group discussions. Because of the way in which the focus group discussions are recorded, the research team will not be able to withdraw or destroy individual participant responses.

**Additional Costs and Reimbursement:** There are no costs associated with participating in this research project, nor will you be paid. However, you will receive a voucher for $20 for, for example, Coles, to reimburse you for any reasonable travel, parking, meals, and other expenses while completing the interview or focus group.

**Psychological Distress:** You may feel that some of the questions we ask are stressful or upsetting. If you do not wish to answer a question, you may skip it and go to the next question, or you may stop immediately. As the interviews/focus groups will take place at your local community centre or disability service, if necessary, we will be able to bring in your support worker to provide additional support. If you become upset or distressed as a result of your participation in the research project, the research team will be able to arrange for counselling or other appropriate support. Alternatively, several free contactable support services are included at section 9. Any counselling or support will be provided by qualified staff who are not members of the research team. This counselling will be provided free of charge.

1. **What will happen to information about me?**

By signing the consent form, you consent to the research team collecting and using information about you for the research study.

The research team will store the data collected from you for this research project for:

- A minimum of 7 years after the completion of the research.

The information about you will be stored in a:

- Re-identifiable format where any identifiers such as your name, address, date of birth will be replaced with a unique code.
- Information collected from you in an electronic format stored on a UNSW password protected OneDrive only accessible to the approved research investigators.
- Information collected from you using paper-based measures will be stored in the secure office of the Chief Investigator Professor Iva Strnadová at the School of Education, UNSW, and only the approved research investigators will have access to this information.
- Audio or video recordings will be stored on a UNSW password protected OneDrive only accessible to the approved research investigators which will also be made available to a professional transcription service. Recordings will only be made available after a confidentiality agreement has been signed.

The information you provide is personal information for the purposes of the Privacy and Personal Information Protection Act 1998 (NSW). You have the right of access to personal information held about you by the University, the right to request correction and amendment of it, and the right to make a complaint about a breach of the Information Protection Principles as contained in the PPIP Act. Further information on how the University protects personal information is available in the [**UNSW Privacy Management Plan**](https://www.legal.unsw.edu.au/compliance/privacyhome.html).

1. **How and when will I find out what the results of the research study are?**

The research team intend to publish and/ report the results of the research. All Information will be published in a way that will not identify you.

If you would like to receive a copy of the results you can let the research team know by inserting your email or mailing address in the consent form. We will only use these details to send you the results of the research.

1. **What if I want to withdraw from the research study?**

If you do consent to participate, you may withdraw at any time. You can do so by completing the ‘Withdrawal of Consent Form’ which is provided at the end of this document or you can ring the research team and tell them you no longer want to participate. Your decision not to participate or to withdraw from the study will not affect your relationship with UNSW Sydney or NSW Health. you decide to leave the research study, the researchers will not collect additional information from you. You can request that any identifiable information about you be withdrawn from the research project.

1. **What if I have a complaint or any concerns about the research study?**

If you have a complaint regarding any aspect of the study or the way it is being conducted, please contact the UNSW Human Ethics Coordinator:

**Complaints Contact**

| **Position** | UNSW Human Research Ethics Coordinator |
| --- | --- |
| **Telephone** | + 61 2 9385 6222 |
| **Email** | [humanethics@unsw.edu.au](mailto:humanethics@unsw.edu.au) |
| **HC Reference Number** | **HC210342** |

1. **What should I do if I have further questions about my involvement in the research study?**

The person you may need to contact will depend on the nature of your query. If you require further information regarding this study or if you have any problems which may be related to your involvement in the study, you can contact the following member/s of the research team:

**Research Team Contact Details**

| **Name** | Professor Iva Strnadová |
| --- | --- |
| **Position** | Chief Investigator, Professor in Special Education and Disability Studies |
| **Telephone** | 0426 959 172 |
| **Email** | [i.strnadova@unsw.edu.au](mailto:i.strnadova@unsw.edu.au) |

**Chief Investigator**

| **Name** | Dr Elizabeth Emma Palmer |
| --- | --- |
| **Position** | Coordinating Chief investigator, Clinical Lecturer and Clinical Geneticist |
| **Telephone** | 02 93825583 |
| **Email** | Elizabeth.palmer@unsw.edu.au |

**Support Services Contact Details**

If at any stage during the study, you become distressed or require additional support from someone not involved in the research please call:

| **Name/Organisation** | Lifeline |
| --- | --- |
| **Telephone** | 13 11 14 |

| **Name/Organisation** | Mental Health Access Line |
| --- | --- |
| **Telephone** | 1800 011 511 |

| **Name/Organisation** | People with Disability Australia |
| --- | --- |
| **Telephone** | (02) 9370 3100. |

**Consent Form – Participant providing own consent**

**Declaration by the participant**

- I understand I am being asked to provide consent to participate in this research study;
- I have read the Participant Information Sheet, or someone has read it to me in a language that I understand;
- I understand the purposes, study tasks and risks of the research described in the study;
- Recordings: I understand that the research team will audio and or /video record the interviews and /or focus groups; I agree to be recorded for this purpose.
- I provide my consent for the information collected about me to be used for the purpose of this research study only.
- I have had an opportunity to ask questions and I am satisfied with the answers I have received;
- I freely agree to participate in this research study as described and understand that I am free to withdraw at any time during the study and withdrawal will not affect my relationship with any of the named organisations and/or research team members;
- I would like to receive a copy of the study results via email or post, I have provided my details below and ask that they be used for this purpose only;
- I understand that I will be given a signed copy of this document to keep.
- I understand that the results of the research will be made available on the Disability Innovation Institute, UNSW, website.
- I would like to receive a copy of the study results via email or post, I have provided my details below and ask that they be used for this purpose only.

**Name: _____________________________________**

**Address: ___________________________________**

**Email Address: ______________________________**

**Participant Signature**

| Name of Participant (please print) |  |
| --- | --- |
| Signature of Research Participant |  |
| Date |  |

**Declaration by Researcher***

- I have given a verbal explanation of the research study; its study activities and risks and I believe that the participant has understood that explanation.

**Researcher Signature***

| Name of Researcher (please print) |  |
| --- | --- |
| Signature of Researcher |  |
| Date |  |

**^+^An appropriately qualified member of the research team must provide the explanation of, and information concerning the research study.**

**Note: All parties signing the consent section must date their own signature.**

**Form for Withdrawal of Participation**

I wish to **WITHDRAW** my consent to participate in this research study described above and understand that such withdrawal **WILL NOT** affect my relationship with The University of New South Wales.

- I am withdrawing my consent and I would like any identifiable information collected about me which I have provided for the purpose of this research study withdrawn.
- I am withdrawing my consent to participate in further components of this research and provide my permission for the research team to retain and/or use information collected about me which I have provided for the purpose of this research.
- I am withdrawing my consent and I understand that any information already published and/or not linked to my identity cannot be withdrawn from the research.

**Participant Name**

| Name of Participant  (please type) |  |
| --- | --- |
| Date |  |

**The section for Withdrawal of Participation should be forwarded to:**

| CI Name: | Dr Elizabeth Emma Palmer |
| --- | --- |
| Email: | Elizabeth.palmer@unsw.edu.au |
| Phone: | 02 93825583 |
| Postal Address: | Level 9, Bright Alliance Building, School of Women’s and Children’s Health, Cnr of Avoca and High Street, Randwick, NSW, Australia |

**
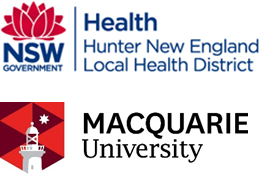

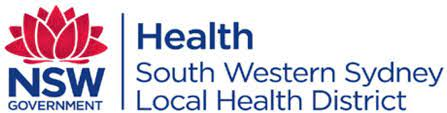
**
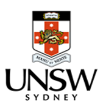


**PARTICIPANT INFORMATION STATEMENT AND CONSENT FORM**

**GeneEQUAL**


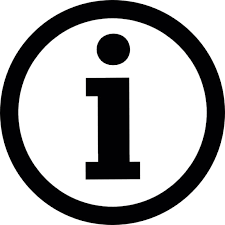
This sheet has information about a research study called **GeneEQUAL.**

**
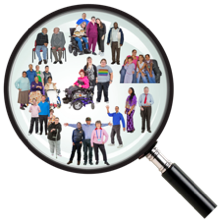
Research** means finding out what people think about things and using the information to help other people.


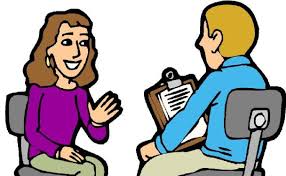


You can ask someone you know and trust to help you understand this sheet.

**Who is doing the research?**


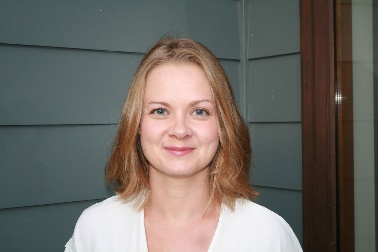


Dr Emma Palmer.

I am a researcher at the University of New South Wales.


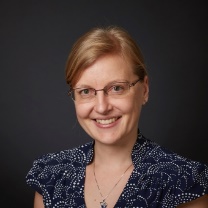


Professor Iva Strnadová.

I am a researcher at the University of New South Wales.


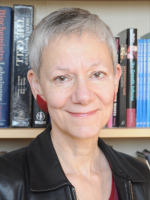


Professor Jackie Leach Scully.

I am a researcher at the University of New South Wales.


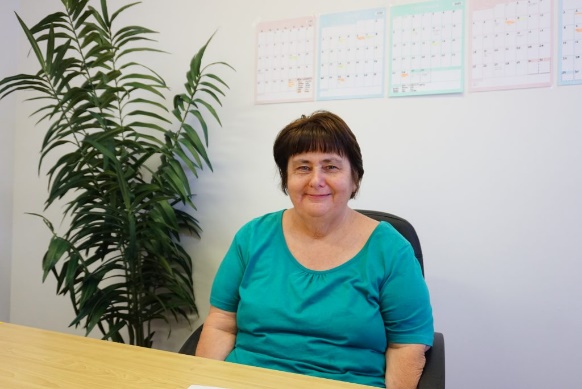


Ms Julie Loblinzk.

I am working at Self Advocacy Sydney Inc.

I am also a researcher at the University of New South Wales.


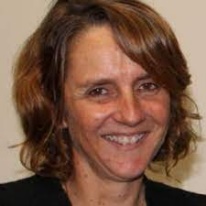


Mrs Jackie Boyle.

I work in NSW Health

**What do we want to find out?**


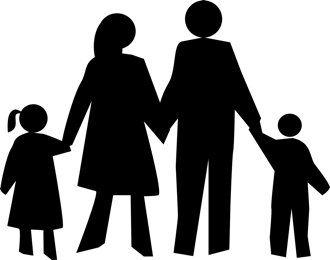


We want to learn about what **you** think about:


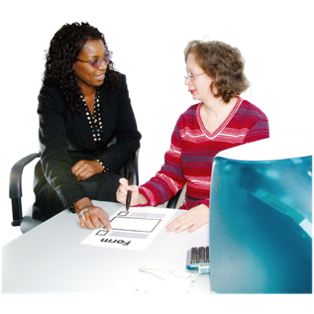


**Genetic counselling**.

Genetic counselling means talking about why people have a health or learning difficulty.


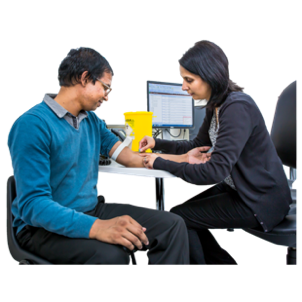


**Gene testing.**

Gene testing means a blood or spit test to help find out why people have a health or learning difficulty.

**What does taking part involve?**


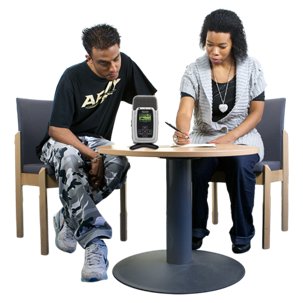
Taking part in the study will involve meeting one or more of us to talk. We will ask you about:

- What it was like to have genetic counselling or testing
- How you think genetic testing or counselling should be done


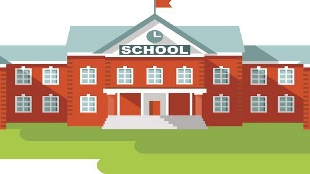


We will meet you at a place you choose.


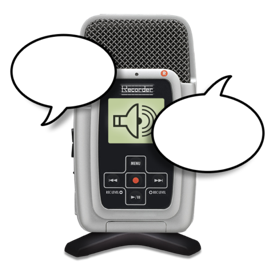


We will ask to record what you say.

If you do not want us to, that is OK.


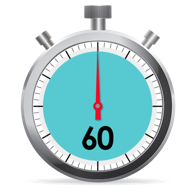
You can either talk to a researcher one-on-one, or you can decide to take part in a focus group.

You can take part face-to-face, via phone call or online. If you prefer to talk to us online, we will use your preferred platform (Zoom, Skype, Facetime, Teams, etc.).

Talking one-on-one will take about 60 minutes.

A focus group will take about 60 minutes.


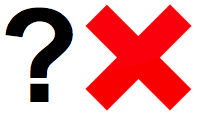


You can choose which questions to answer.

You can say no if you do not want to answer a question.


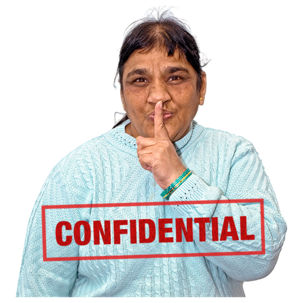
We will keep your personal information private.

We will not tell anybody your name or where you live.

No one will know it was you who took part.

We will write about what we find out.


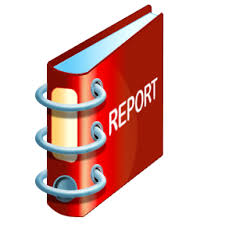


If you want, we will send you a summary of what we found out.

If you need help to understand the summary or want to learn about the findings in more detail, the researchers will meet you.


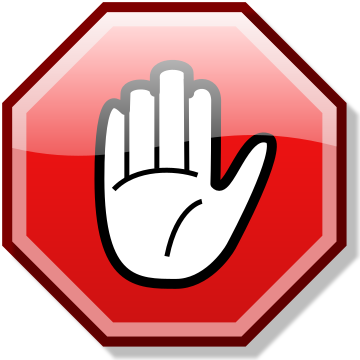
You only have to take part if you want to. You can say no. It’s your choice. No one will be angry if you decide you do not want to do it.

Even if you say yes, you can change your mind later.

If you want to take part, please sign the consent form.


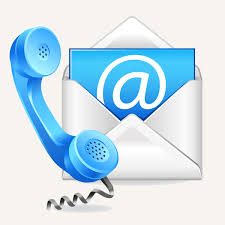


These are some people you can get in touch with if you need to.


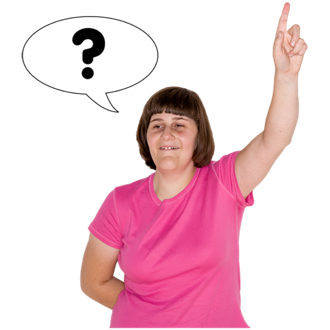


If you have any questions or want more information you can get in touch with the chief researchers:

Dr Emma Palmer

Phone: 02 93825583

Email:elizabeth.palmer@unsw.edu.au


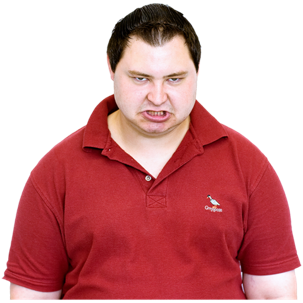
If you want to complain about the study, can you get in touch with:

Human Research Ethics Coordinator
Phone: (02) 9385 6222
Email: [humanethics@unsw.edu.au](mailto:humanethics@unsw.edu.au)

It is their job to listen to you and find out what happened. Tell them this number: **HC210342**


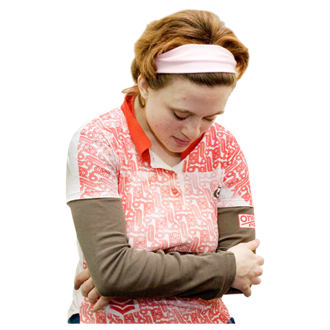


If you feel upset after taking part, you can talk to some who supports you or call:

Mental Health Access Line: 1800 011 511

Lifeline: 13 11 14

People with Disability Australia: (02)9370 3100.

Before you sign the consent form, please check:


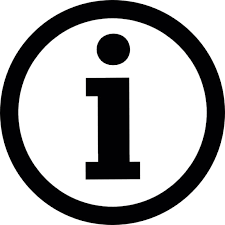
I have read the sheet or had someone read it to me so I understand.

I understand what will happen during the study and why it is happening.


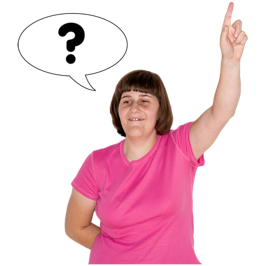


I was allowed to ask questions about what will

happen.

I was happy with the answers I was given.


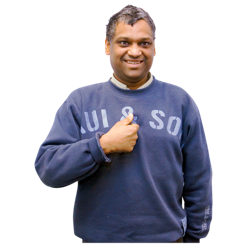


I choose to take part in the study.

No one is making me.


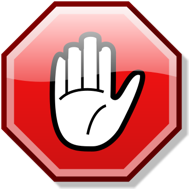


I know that I can change my mind at any time.


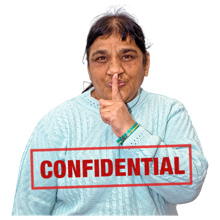


I know that Iva and Julie will collect information about me, but that they will keep my information private.

No one will know it was me who took part.


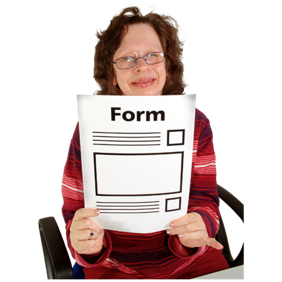


I know that I will be given a copy of this form to keep.

**GeneEQUAL**


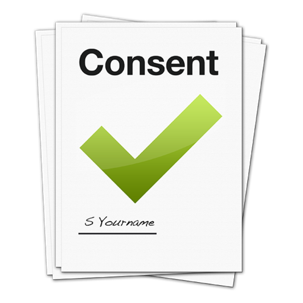
**I WANT TO TAKE PART**

I am signing this form because I understand about the research and I want to take part.

My signature: ________________________________

Date: ________________________________

My phone number: ________________________________

My address: ________________________________

My email address: ________________________________

I understand that you will only use my contact details for me to take part in the research and to tell me about the findings afterwards.

Witness signature: ________________________________

Date: ________________________________

A witness is someone who saw you sign the form.

**GeneEQUAL**

**I DON’T WANT TO TAKE PART ANYMORE**


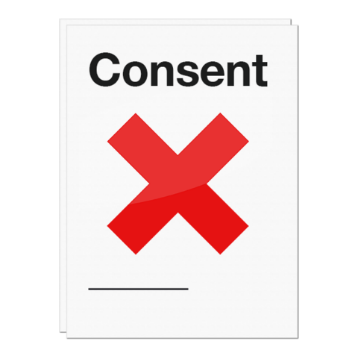


I am signing this form because I changed my mind.

I don’t want to take part anymore and I don’t want you to use what I said.

My signature: _____________________

Date: _____________________

Witness signature: _____________________

Date: _____________________

A witness is someone who saw you sign the form.

**Send this form to:**

Dr Elizabeth Emma Palmer

Level 9, Bright Alliance Building, School of Women’s and Children’s Health,

Cnr of Avoca and High Street,

Randwick,

NSW,

Australia
