# Supplementary material for: “I am not a number!” Opinions and preferences of people with intellectual disability about genetic healthcare
Source: Eur J Hum Genet. 2023 Jan 20;31(9):1057–65. doi: 10.1038/s41431-023-01282-3 (PMC10474088; doi:10.1038/s41431-023-01282-3)
Supplement: Supplementary file 2 — Appendix B [file 41431_2023_1282_MOESM2_ESM.docx]

**Project name: GeneEQUAL**

**INTERVIEW/ FOCUS GROUP PROTOCOL**

Welcome and acknowledgement of country.

Explanation of ‘ground rules’ of focus group/ interview:

- A safe space, people should feel free to say what they feel, there is no right or wrong answers.
- We will all be supportive and kind, and not attack what another person says.
- If anyone is feeling sad or worried to let us know and we can offer them support or time out.
- There will be follow up available after the session if there are any questions or worries.

The session will start by explaining what the interview/ focus group is about, and how we are hoping to gather information about what they know about the cause of their learning difficulties:

- If they know if they have a particular genetic condition (such as Down syndrome, Prader-Willi syndrome), or
- What they know about genes and genetic testing, their thoughts and opinions on genetic testing, and
- If they or their family members have had genetic testing, what was their experience (e.g., what went well, what did not go well and what would they change about the experience).

It will be emphasised that:

- If any questions make the participant feel uncomfortable, they can ask to stop at any time they like, just by letting the facilitator know.
- They do not have to answer any question that they do not want to.
- What they tell us will be kept private – that is we will not link what they say to their name or anything that could identify who they are.

**Demographic questions**

1. We will use your first name if you want. If you prefer, choose another name we should use to keep what you tell us private.
2. How old are you?
3. Are you:

- Single?
- Dating (courting)?
- Married?
- Widowed?
- Living with a partner?
- Separated/ divorced / widowed?

1. Do you have any children?

If yes, how many?

How old are they?

1. Where do you live?

- Family house ?
- Group home?
- Apartment?
- Institution?
- Supported living?
- On your own with support?
- On your own without support?
- Nursing home?
- Residential care?

1. Do you live on your own?

If not, who lives with you?

For example: Do you live with your children? Do you live with your partner? Parents? Roommate(s)? Friends? People you have not chosen to live with?

1. Do you work? What kind of work did/do you do? Do you work full-time/ part-time? Paid or volunteering? Casual paid work/ freelance/ consultancy?
2. Are you an Aboriginal/ a Torres Strait Islander?
3. Do you live in the city or in a rural, or remote area? Please what is your postcode?

**Experiences with genetic counselling or testing - before the visit**

Thank you for taking part in this interview/ focus group. I understand that you went to visit a genetics doctor in the past. Do you remember why you went to see them?

- [PROMPTS: Your doctor or parent suggested, you were pregnant, you were planning a baby, you had had a gene test result, you wanted to know more about your health and learning, you had a health problem (like breast cancer)]
- [PROMPTS: Whose idea was it to see a genetics team?]

**Genetics clinic appointment**

Can you tell me more about where you saw the genetics team and how that went?

- [PROMPTS: Was it at home, in a hospital, in a clinic, in a community centre]
- [PROMPTS: Who came with you? Was that good? Who would you like to have been there?]

Can you tell me what you remember talking about?

- [PROMPTS: – why you had learning difficulties, genes, chromosomes, gene tests, blood tests, health checks]

**Understanding of genetics**

Have you heard of genes or chromosomes?

***>>>If participant answers yes***

Tell me what you know about genes or chromosomes?

- [PROMPTS: who told you about this],
- [PROMPTS: Did you find talking about this in the past helpful? What did you like? What did you not like?]

**Talking about babies/ pregnancies**

Would you like to have babies?

- [PROMPTS: If yes/ no – why/ why not]

What do you think is the chance of you having a baby with learning difficulties?

- [PROMPTS: What do you feel about that?]
- [PROMPTS: Do you think there is a test that you could do before you were pregnant or when you are pregnant to check if the baby will have learning difficulties?]

***>>>If participant answers yes:***

- [PROMPTS: would you choose to have that test – why/ why not? How would you feel if the test showed your baby would have learning difficulties?]

**Gene testing discussion /consent**

What were you told about doing a genetic test?

[PROMPTS: What do you remember being told about?]

[PROBES: uncertain results, incidental findings?]

[PROMPTS: Can you remember if you were told why gene testing might be helpful]

[PROBES: Help you with planning pregnancies, help others in your family, help with your health care? Help access support/ NDIS? Help with scientific research? Trying to understand why you have had health and or learning problems?]

Can you tell me what you can remember about choosing to have the genetic test?

- [PROMPTS: Could you say yes or no? Do you remember any pictures or bits of paper explaining things? Were you asked to read a form…’a consent form’ to say if you agreed to have the test? Who signed the form? How did you feel about this?]

**Gene test result**

What do you remember about the result of the genetic test?

***>>>If the participant remembers that the genetic test provided a diagnosis****:*

- [PROMPTS: Do you remember the name of the condition?]
- [PROMPTS: What can you tell me about this condition?
- [PROMPTS: Do you know anyone else with this condition?]
- [PROMPTS: Can you remember getting any information about the condition?]
- [PROBES: e.g. website, booklet or factsheet?]

How did you feel knowing you have [insert name genetic condition]?

- [PROMPTS**:** What do you like? What don’t you like?]
- [PROMPTS: Has this information been useful to you or your family?]
- [PROMPTS: Is there anything else you would like to know about this condition?]

***>>>If the participant cannot remember is the genetic test provided a diagnosis, or if the result was negative or uncertain.***

How did you feel after the genetic test?

- [PROMPTS: Did the genetic test leave you with more questions?]

**Overall impressions / wrap up**

What would you tell other people with intellectual disability about seeing a genetic team?

- [PROMPTS: A good idea? A bad idea?]
- [PROMPTS If would recommend: What was good? What did you like talking about?]
- [PROMPTS If would not recommend: What did you not like talking about?]

Is there anything the genetic team could have done better to prepare you for the appointment?

How do you think genetic doctors should explain genes and genetic testing to people with intellectual disability/ learning problems?

Is there anything you would like to add to what you have already told us?
